# Novel associations between *MTDH* gene polymorphisms and invasive ductal breast cancer: a case–control study

**DOI:** 10.1007/s12672-024-01086-x

**Published:** 2024-07-09

**Authors:** Yan Huang, Dan Dai, Li Zhu, Xianzhong Qi

**Affiliations:** Department of Pathology, The First People’s Hospital of Linping District, #369 Nanyuan Street Yingbin Road, Linping District, Hangzhou, Zhejiang China

**Keywords:** Breast cancer, Invasive, *MTDH*, Polymorphism, Susceptibility

## Abstract

**Objective:**

To reveal the contributing effects of *MTDH* gene SNPs in the risk of invasive ductal breast cancer (IDC).

**Patients and methods:**

A case–control study was conducted, recruiting a total of 300 cases of IDC and 565 cancer-free controls from East China. Genotyping of three single-nucleotide polymorphisms (SNPs) in the *MTDH* gene was performed. Genomic DNA was extracted from peripheral blood samples of patients. The three SNPs (rs1311 T > C, rs16896059 G > A, rs2449512 A > G) in the *MTDH* gene were selected for detection using a TaqMan real-time polymerase chain reaction assay. The association between *MTDH* and the risk of IDC was analyzed employing an epidemiology case–control study and a multinomial logistic regression model.

**Results:**

Among the three evaluated SNPs, rs1311 T > C, rs16896059 G > A, and rs2449512 A > G demonstrated a significant association with an increased risk of IDC. Furthermore, stratified analysis revealed that individuals carrying the rs1311 CC genotype, rs16896059 GA/AA genotypes, and rs2449512 GG genotype were more susceptible to developing IDC in subgroups of patients younger than 53 years, without family history of IDC, pre-menopause status, clinical stage 2, high grade, with no distant metastasis or invasion, Her2-positive type, ER positive, PR positive, and Ki67 cells less than 10%. However, carriers of the rs16896059 GA/AA genotypes and rs2449512 GG genotype had an elevate the risk of IDC in patients with tumor size larger than 2 cm, post-menopause status, clinical stage 3, with invasion, lymph node infiltration, ER negative, PR negative, Her2 negative, and Ki67 cells exceeding 10%. Compared to the reference haplotype TGA, haplotypes TAA, TAG, and TGG were significantly associated with an increased IDC risk.

**Conclusion:**

In this study, we demonstrated a significant association between *MTDH* gene polymorphisms and an increased risk of IDC. Moreover, our findings suggested that *MTDH* gene polymorphisms could serve as a potential biomarker for IDC subtyping and therapeutic selection.

## Introduction

Recent research indicates that breast cancer has emerged as the most prevalent malignant tumor, ranking second only to lung cancer as the leading cause of cancer-related death among women worldwide [1]. The incidence of breast cancer continues to escalate annually, although the prevalence varies significantly among countries due to differences in age and lifestyle factors [2]. Breast cancer is histopathologically classified into several subtypes, with invasive ductal carcinoma (IDC) being the most common subtype accounting for approximately 70–80% of cases. Based on the expression of estrogen receptor (ER), progesterone receptor (PR), and human epidermal growth factor receptor (Her2), breast cancer can be categorized into four molecular subgroups: luminal A type, luminal B type, Her2-positive type, and basal-like type [3]. Various factors such as family history, breastfeeding, obesity, and environmental influences, contribute to the risk of developing breast cancer. Additionally, genetic variations play a crucial role in both the initiation and progression of breast cancer [4]. Single nucleotide polymorphism (SNP) represents one form of genetic variation that has been extensively studied regarding its association with susceptibility to breast cancer development [5].

SNPs in metastasis-related genes function as vital roles in the carcinogenesis of breast cancer, as demonstrated by various studies. The association between the variations of these genes and breast cancer has been corroborated by research, such as the identification of the polymorphism of tissue inhibitor of metalloproteinase-2 (*TIMP-2*) as a risk factor for breast cancer [6]. Li Z, et al. also reported a link between the vascular endothelial growth factor (*VEGF*) gene − 634G/C polymorphism and an increased risk of breast cancer [7]. Moreover, SNPs in *ERBB3* and *BARD1* genes were found to indicate a poorer prognosis for HER2-positive breast cancer patients [8]. The *NF-κB1* rs28362491 polymorphism was associated with an increased risk of breast cancer in Lower Northern Thailand [9], while a positive association between *NF-κB* rs3774937 and breast cancer was observed in the Middle Eastern-North African population [10]. Alanazi MS, et al. investigated the association of Wnt signaling pathway gene polymorphisms with breast cancer and found that the SNP in beta-catenin was positively related to the risk of breast cancer in Saudi patients [11]. Collectively, these findings corroborate the notion that polymorphisms of metastasis-related genes are associated with the risk of breast cancer.

Metadherin (*MTDH*), also known as astrocyte elevated gene 1 (*AEG-1*), is located at chr8q22.1 and functions as a metastatic adhesion protein, making it a therapeutic target in various cancers [12]. MTDH is overexpressed in multiple cancers, including breast cancer [13]. A close relationship has been observed between MTDH expression and the poor prognosis of breast cancer patients [14]. The upregulation of MTDH is associated with a better prognosis of Her2-positive breast cancer patients [15]. MTDH promotes metastasis and invasion by interacting with VEGF [16], Twist1 [17], NF-κB [18], and other genes involved in metastasis-related signaling pathways. Inhibition of MTDH reduces paclitaxel resistance in breast cancer cells [19]. Moreover, suppressing MTDH activity hinders the metastatic potential of breast cancer cells [20]. To date, only one study has revealed a negative association between *MTDH* (− 470G > A) polymorphism and ovarian cancer susceptibility [21]. Given that numerous metastasis-related gene polymorphisms are known to be associated with an increased risk of breast cancer, the impact of *MTDH* polymorphisms on breast cancer susceptibility has not yet been reported.

In the current investigation, a total of three SNPs were selected to evaluate the association between *MTDH* polymorphisms and IDC. The study was designed as a case–control analysis, utilizing samples from Eastern China.

## Materials and methods

### Study subjects and data collection

A sample of 300 breast cancer patients and 565 age-matched and ethnicity-matched healthy controls from Eastern China, aged 24 to 96 years old, median age was 53, was recruited from The First People’s Hospital of Linping District, Hangzhou City, Zhejiang Province. This study collected breast cancer cases from January 2013 to May 2020. The required sample size was estimated based on a similar study [22]. Cases were selected according to pathological diagnosis, while controls were recruited from health adult women undergoing physical examination at The First People’s Hospital of Linping District, Zhejiang Province. Exclusion criteria included women with other malignancies, gynecological diseases, endocrine system disorders, or who were breastfeeding.

The comprehensive clinical and biological features of breast cancer patients, including age, clinical stage, tumor size, pathological grade, family history, molecular type, lymph node involvement, invasion, and metastasis were comprehensively analyzed and tabulated in Table 1.

**Table 1:** Characteristics of the participants

	Cases (300)	Controls (565)	*p*
Age (y)	53.6 ± 11.7	52.3 ± 12.6	0.126
BMI (kg/m2)	23.1 ± 4.8	22.8 ± 4.2	0.246
Family history			0.001
Yes	18 (6.0%)	10 (1.8%)	
No	282 (94.0%)	555 (98.2)	
Menopausal age (y)	11.6 ± 2.4	11.9 ± 1.8	0.425
Pausimenia			0.236
Post-menopause	178 (59.3%)	248 (40.3%)	
Pre-menopause	132 (40.7%)	217 (59.7%)	
Tumor size			
< 2 cm	174 (58.0%)		
≥ 2 cm	126 (42.0%)		
Clinical stage			
1	24 (8.0%)		
2	183 (61.0%)		
3	93 (31.0%)		
Pathological grade			
Low	290 (96.7%)		
High	10 (3.3%)		
Distance metastasis			
Yes	11 (3.6%)		
No	289 (96.4%)		
Invasion			
Yes	238 (79.3%)		
No	62 (21.7%)		
Node infiltration			
Yes	102 (34.0%)		
No	198 (67.0%)		
Molecular type			
LuminalA	123 (41.0%)		
LuminalB	82 (27.3%)		
Her2-positive	47 (15.7%)		
B asal-like	48 (16.0%)		
ER expression			
Negative	93 (31.0%)		
Positive	207 (69.0%)		
PR expression			
Negative	95 (31.7%)		
Positive	205 (68.3%)		
Her2 expression			
Negative	172 (57.3%)		
Positive	128 (42.7%)		
Ki67 expression			
< 10%	219 (73.0%)		
≥ 10%	81 (27.0%)		

The study was granted ethical approval by the Ethics Committee of The First People’s Hospital of Linping District, Zhejiang Province (reference number: 2018-152), and written informed consent was obtained from all samples enrolled in accordance with the Declaration of Helsinki.

### MTDH SNPs selection and genotyping

The NCBI dbSNP database (http://www.ncbi.nlm.nih.gov/projects/SNP) and SNPinfo (http://snpinfo.niehs.nih.gov/snpfunc.htm) online software were utilized to identify selected potentially functional SNPs. The selection criteria were based on published studies, as follows [23, 24]: the minor allele frequency (MAF) of SNPs identified in HapMap no less than 5% for Chinese Han subjects; SNPs were located in the 5ʹ and 3ʹ untranslated region, exons and the junctions of exon and intron of the *MTDH* gene, as well as in regions with low linkage disequilibrium (R2 < 0.8). Three SNPs (rs1311 T > C, rs16896059 G > A, rs2449512 A > G) in the *MTDH* gene were selected. Rs1311 and rs2449512 are situated in the 3ʹUTR of *MTDH*, potentially serving as binding sites for miRNAs. rs16896059, on the other hand, is located in the promoter of *MTDH* and is predicted to be a binding site for transcriptional factors. A quantity of 1 μg genomic DNA was extracted from IDC patients’ and control samples’ peripheral blood. The reaction system and conditions of the Taqman real-time PCR assay were in accordance with a published reference [25]. Taqman probes of rs1311 (Assay ID; C_11331064_30), rs16896059 (Assay ID: C_27850029_10), and rs2449512 (Assay ID: C_15799756_10) were purchased from Thermo Fisher. To ensure the accuracy of the genotyping results, 10% of the samples were randomly selected for genotyping by DNA sequencing. A concordance rate of 100% was achieved for the quality control samples.

### Statistical analysis

The goodness-of-fit χ^2^ test was employed to assess whether the znalyzed SNPs in *MTDH* gene deviated from Hardy–Weinberg equilibrium (HWE) among controls. A two-sided χ^2^ test was conducted to compare demographic variables and genotype frequencies of patients and controls. Odds ratios (ORs), age-adjusted ORs, and their corresponding 95% confidence intervals (CIs) for the association between SNPs and susceptibility of breast cancer were counted using unconditional logistic regression analyses. Furthermore, a combination of rs1311 T > C, rs16896059 G > A, and rs2449512 A > G was considered a haplotype. Unphased genotype data were utilized to determine haplotype frequencies and individual haplotypes. Logistic regression analysis also assisted in calculating haplotype frequencies and distinct haplotypes, with adjustment for age. The haplotype with the highest frequency was used as the reference group to calculate ORs for haplotypes associated with IDC risk [26]. All statistical analyses were conducted through the SAS statistical package (version 9.1; SAS Institute, Cary, NC). All P values in this study were two-sided, and a P value < 0.05 was supposed to be statistically significant.

## Results

### Population characteristics

The summarized information of the demographic and clinical features of IDC patients and healthy controls was listed in Table 1. No significant differences were observed between Eastern Chinese women with IDC and the controls in terms of age (P = 0.1263), BMI (P = 0.2457), and menopausal age (P = 0.4251). However, a significant difference was noted between the premenopausal women who had IDC compared to the control group (P = 0.236). Among IDC cases, tumors less than 2 cm accounted for 58% (174 cases) while those greater than or equal to 2 cm accounted for 42% (126 cases). Regarding clinical stage, there were 24 cases (8%) at stage1183 cases (61%) at stage 2, and 93 cases (31%) at stage 3. In terms of pathological grade, high-grade tumors were present in three patients (3.3%), while low-grade tumors were present in 96.7% (290 cases). Eleven patients (3.6%) had distant metastasis, while 96.4% (289 cases) did not. Of the 208 IDC cases (79.3%) analyzed, 62 cases (21.7%) had no invasion. One hundred and two cases (34.0%) had node infiltration, while 198 cases (67.0%) did not. Regarding molecular subtype, 41.0% (123 cases) were luminal A type, 27.3% (82 cases) were luminal B type, 15.7% (47 cases) were Her2-positive type, and 16.0% (48 cases) were basal-like type. ER was expressed in 31.0% (207) and negatively expressed in 69.0% (93) cases. PR was expressed in 68.3% (205) and negatively expressed in 31.7% (95) cases. The expression of Her2 was positive in 42.7% (128 cases) and negative in 57.3% (172 cases). Lastly, 27.0% (81 cases) had more than 10% positive Ki67 expressed cells, while 73.0% (219 cases) had less than 10% positive Ki67 expressed cells in IDC tissues.

### Association of *MTDH* gene polymorphisms with IDC risk

The genotype frequencies of *MTDH* associated with IDC risk were presented in Table 2. In the single-locus analysis, carriers of the rs1311 (CC vs. TT: adjusted OR = 2.775, 95% CI 1.114–6.910, P = 0.0283), rs16896059 (GA vs. GG: adjusted OR = 1.916, 95% CI 1.114–3.295, P = 0.0187; AA vs. GG: adjusted OR = 31.656, 95% CI 4.155–241.196, P = 0.0009), and rs2449512 (GG vs. AA: adjusted OR = 4.504, 95% CI 2.093–9.691, P = 0.0001) variant alleles were found to contribute to an elevated risk of IDC.

**Table 2:** Logistic regression analysis of associations between MTDH polymorphisms and IDC susceptibility

Genotype	Cases	Controls	*p*^a^	Crude OR	*p*	Adjusted OR	*p*^b^
	(N = 300)	(N = 565)		(95% CI)		(95% CI)^b^	
rs1311 T > C (HWE = 0.642)							
TT	244 (81.33)	448 (79.29)		1.00		1.00	
TC	43 (14.33)	109 (19.29)		0.724 (0.492–1.065)	0.1014	0.710 (0.479–1.052)	0.0875
CC	13 (4.33)	8 (1.42)		2.981 (1.219–7.290)	0.0167	2.775 (1.114–6.910)	0.0283
Additive	69	117	0.0078	1.040 (0.774–1.397)	0.7938	1.013 (0.750–1.368)	0.9315
Dominant	56 (18.67)	117 (20.71)	0.4750	0.879 (0.616–1.253)	0.4752	0.856 (0.596–1.229)	0.3992
Recessive	287 (95.67)	557 (98.58)	0.0080	3.151 (1.291–7.689)	0.0117	2.944 (1.185–7.313)	0.0200
rs16896059 G > A (HWE = 0.403)							
GG	254 (84.67)	534 (94.51)		1.00		1.00	
GA	29 (9.67)	30 (5.31)		2.032 (1.194–3.459)	0.0090	1.916 (1.114–3.295)	0.0187
AA	17 (5.67)	1 (0.18)		35.733 (4.730–269.948)	0.0005	31.656 (4.155–241.196)	0.0009
Additive	63	32	< 0.0001	2.933 (1.961–4.387)	< 0.0001	2.772 (1.839–4.178)	< 0.0001
Dominant	46 (15.33)	31 (5.49)	< 0.0001	3.120 (1.932–5.038)	< 0.0001	2.902 (1.781–4.730)	< 0.0001
Recessive	283 (94.33)	564 (99.82)	< 0.0001	33.873 (4.486–255.775)	0.0006	30.116 (3.953–229.426)	0.0010
rs2449512 A > G (HWE = 0.103)							
AA	221 (73.67)	449 (79.47)		1.00		1.00	
AG	58 (19.33)	105 (18.58)		1.122 (0.784–1.607)	0.5287	1.081 (0.749–1.560)	0.6757
GG	21 (7.00)	11 (1.95)		3.879 (1.838–8.187)	0.0004	4.504 (2.093–9.691)	0.0001
Additive	100	127	0.0007	1.477 (1.136–1.921)	0.0036	1.501 (1.148–1.964)	0.0030
Dominant	79 (26.33)	116 (20.53)	0.0519	1.384 (0.997–1.921)	0.0524	1.374 (0.983–1.920)	0.0633
Recessive	279 (93.00)	554 (98.05)	0.0002	3.791 (1.802–7.973)	0.0004	4.436 (2.068–9.515)	0.0001

^a^*χ*^*2*^ test for genotype distributions between breast cancer cases and controls

^b^Adjusted for age

### Stratification analysis of identified SNPs

The influence of the selected *MTDH* polymorphisms (rs1311 T > C, rs16896059 G > A, rs2449512 A > G) on specific subtypes of IDC was further examined (Table 3). For rs1311 T > C, a significantly increased risk effect was observed among patients aged younger than 53 years (adjusted OR = 7.997, 95% CI 1.527–41.880, P = 0.0139), with tumor size less than 2 cm (adjusted OR = 3.554, 95% CI 1.323–9.548, P = 0.0119), no family history (adjusted OR = 2.794, 95% CI 1.106–7.061, P = 0.0298), pre-menopause (adjusted OR = 4.609, 95% CI 1.587–13.386, P = 0.0050), clinical stage 2 (adjusted OR = 3.187, 95% CI 1.183–8.588, P = 0.0219), high pathological grade (adjusted OR = 1.052, 95% CI 1.228–7.586, P = 0.0163), no distance metastasis (adjusted OR = 3.058, 95% CI 1.233–7.584, P = 0.0159), no invasion (adjusted OR = 2.700, 95% CI 1.037–7.026, P = 0.0419), no node infiltration (adjusted OR = 2.837, 95% CI 1.065–7.588, P = 0.0370), luminal B type (adjusted OR = 4.268, 95% CI 1.312–13.887, P = 0.0159), Her2-positive type (adjusted OR = 4.402, 95% CI 1.118–17.339, P = 0.0341), ER positively expressed (adjusted OR = 2.803, 95% CI 1.043–7.530, P = 0.0410), PR positively expressed (adjusted OR = 2.831, 95% CI 1.053–7.609, P = 0.0391), Her2 positively expressed (adjusted OR = 4.402, 95% CI 11.590–12.193, P = 0.0043), and Ki67 expressed cells < 10% (adjusted OR = 2.923, 95% CI 1.117–7.653, P = 0.0289).

**Table 3:** Stratification analysis of MTDH polymorphisms with IDC susceptibility

Variables	rs1311 (cases/controls)		Adjusted OR^a^	*p*^a^	rs16896059 (cases/controls)		Adjusted OR^a^	*p*^a^	rs2449512 (cases/controls)		Adjusted OR ^a^	*p* ^a^
	(cases/controls)		(95% CI)		(cases/controls)		(95% CI)		(cases/controls)		(95% CI)	
	TT/TC	CC			GG	GA/AA			AA/AG	GG		
Age, years												
< 53	94/301	5/2	7.997 (1.527–41.880)	0.0139	86/290	13/13	3.372 (1.507–7.546)	0.0031	90/294	9/9	3.267 (1.259–8.477)	0.0150
≥ 53	193/256	8/6	1.769 (0.604–5.181)	0.2985	168/244	33/18	2.663 (1.451–4.886)	0.0016	189/260	12/2	8.251 (1.826–37.289)	0.0061
Tumor size												
< 2 cm	165/557	9/8	3.554 (1.323–9.548)	0.0119	156/534	18/31	1.827 (0.984–3.393)	0.0563	160/554	14/11	5.770 (2.495–13.348)	< 0.0001
≥ 2 cm	122/557	4/8	1.909 (0.556–6.549)	0.3041	98/534	28/31	4.562 (2.597–8.015)	< 0.0001	119/554	7/11	3.239 (1.203–8.724)	0.0201
Family history												
Yes	17/557	1/8	4.417 (0.516–37.849)	0.1753	16/534	2/31	2.246 (0.491–10.264)	0.2967	16/554	2/11	5.987 (1.208–29.667)	0.0284
No	270/557	12/8	2.794 (1.106–7.061)	0.0298	238/534	44/31	2.952 (1.799–4.845)	< 0.0001	263/554	19/11	4.375 (2.003–9.555)	0.0002
Pausimenia												
Post-menopause	162/557	6/8	1.584 (0.503–4.987)	0.4315	143/534	25/31	2.362 (1.250–4.466)	0.0082	157/554	11/11	8.180 (2.008–33.327)	0.0034
Pre-menopause	125/557	7/8	4.609 (1.587–13.386)	0.0050	111/534	21/31	3.718 (2.017–6.855)	< 0.0001	122/554	10/11	3.380 (1.387–8.240)	0.0074
Clinical stage												
1	24/557	0/8	< 0.001 (< 0.001, > 999.999)	0.9861	23/534	1/31	0.689 (0.090–5.295)	0.7200	22/554	2/11	5.648 (1.139–28.004)	0.0340
2	174/557	9/8	3.187 (1.183–8.588)	0.0219	159/534	24/31	2.507 (1.408–4.462)	0.0018	170/554	13/11	4.699 (2.002–11.027)	0.0004
3	89/557	4/8	2.843 (0.828–9.756)	0.0968	72/534	21/31	4.595 (2.489–8.484)	< 0.0001	87/554	6/11	4.117 (1.454–11.660)	0.0077
Pathological grade												
Low	10/534	0/31	< 0.001 (< 0.001, > 999.999)	0.9863	8/534	2/31	4.084 (0.826–20.195)	0.0845	10/554	0/11	< 0.001 (< 0.001, > 999.999)	0.9844
High	277/534	13/31	3.052 (1.228–7.586)	0.0163	246/534	44/31	2.850 (1.741–4.667)	< 0.0001	269/554	21/11	4.621 (2.152–9.922)	< 0.0001
Distance metastasis												
Yes	11/557	0/8	< 0.001 (< 0.001, > 999.999)	0.9895	5/534	6/31	17.478 (4.916–62.144)	< 0.0001	11/554	0/11	< 0.001 (< 0.001, > 999.999)	0.9883
No	276/557	13/8	3.058 (1.233–7.584)	0.0159	249/534	40/31	2.591 (1.569–4.278)	0.0002	268/554	21/11	4.559 (2.128–9.766)	< 0.0001
Invasion												
Yes	59/557	3/8	3.161 (0.788–12.681)	0.1044	44/534	18/31	6.040 (3.083–11.834)	< 0.0001	53/554	9/11	10.516 (3.929–28.143	< 0.0001
No	228/557	10/8	2.700 (1.037–7.026)	0.0419	210/534	28/31	2.220 (1.288–3.826)	0.0041	226/554	12/11	3.338 (1.422–7.835)	0.0056
Node infiltration												
Yes	97/557	4/8	2.761 (0.789–9.659)	0.1121	74/534	27/31	5.652 (3.154–10.128)	< 0.0001	92/554	9/11	6.128 (2.367–15.866)	0.0002
No	189/557	9/8	2.837 (1.065–7.558)	0.0370	179/534	19/31	1.734 (0.947–3.173)	0.0745	186/554	12/11	3.886 (1.653–9.136)	0.0019
Molecular type												
Luminal A	119/557	4/8	1.837 (0.536–6.290)	0.3331	104/534	19/31	2.839 (1.529–5.269)	0.0009	114/554	9/11	4.851 (1.910–12.322)	0.0009
Luminal B	77/557	5/8	4.268 (1.312–13.887)	0.0159	71/534	11/31	2.416 (1.143–5.106)	0.0208	80/554	2/11	1.795 (0.374–8.609)	0.4644
Her2-positive	44/557	3/8	4.402 (1.118–17.339)	0.0341	37/534	10/31	4.509 (2.046–9.939)	0.0002	43/554	4/11	5.005 (1.514–16.537)	0.0083
Basal-like	47/557	1/8	1.269 (0.153–10.536)	0.8254	42/534	6/31	2.253 (0.880–5.769)	0.0903	42/554	6/11	9.733 (3.240–29.243)	< 0.0001
ER expression												
Negative	89/557	4/8	2.843 (0.828–9.756)	0.0968	77/534	16/31	3.397 (1.764–6.542)	0.0003	83/554	10/11	6.871 (2.775–17.014)	< 0.0001
Positive	198/557	9/8	2.803 (1.043–7.530)	0.0410	177/534	30/31	2.642 (1.536–4.543)	0.0004	196/554	11/11	3.550 (1.471–8.570)	0.0048
PR expression												
Negative	91/557	4/8	2.783 (0.812–9.545)	0.1036	79/534	16/31	3.313 (1.722–6.372)	0.0003	85/554	10/11	6.686 (2.704–16.531)	< 0.0001
Positive	196/557	9/8	2.831 (1.053–7.609)	0.0391	175/534	30/31	2.672 (1.553–4.597)	0.0004	194/554	11/11	3.599 (1.490–8.695)	0.0044
Her2 expression												
Negative	167/557	5/8	1.717 (0.545–5.412)	0.3562	147/534	25/31	2.662 (1.508–4.699)	0.0007	157/554	15/11	5.924 (2.595–13.524)	< 0.0001
Positive	120/557	8/8	4.402 (1.590–12.193)	0.0043	107/534	21/31	3.176 (1.740–5.795)	0.0002	122/554	6/11	2.922 (1.034–5.254)	0.0430
Ki67 expression												
< 10%	209/557	10/8	2.923 (1.117–7.653)	0.0289	186/534	33/31	2.863 (1.687–4.860)	< 0.0001	209/554	10/11	3.045 (1.240–7.475)	0.0151
≥ 10%	78/557	3/8	2.450 (0.627–9.571)	0.1976	68/534	13/31	3.018 (1.494–6.097)	0.0021	70/554	11/11	9.013 (3.677–22.094)	< 0.0001

^a^ Adjusted for age

The rs16896059 polymorphism displayed a more substantial risk association among patients aged < 53 years (adjusted OR = 3.372, 95% CI 1.507–7.546, P = 0.0031), ≥ 53 years (adjusted OR = 2.663, 95% CI 1.451–4.886, P = 0.0016), with tumor size ≥ 2 cm (adjusted OR = 4.562, 95% CI 2.597–8.015, P < 0.0001), no family history (adjusted OR = 2.952, 95% CI 1.799–4.845, P < 0.0001), post-menopause (adjusted OR = 2.362, 95% CI 1.250–4.466, P = 0.0082) and pre-menopause (adjusted OR = 3.718, 95% CI 2.017–6.855, P < 0.0001), clinical stage 2 (adjusted OR = 2.507, 95% CI 1.408–4.462, P = 0.0018) and clinical stage 3 (adjusted OR = 4.595, 95% CI 2.489–8.484, P < 0.0001), high pathological grade (adjusted OR = 2.850, 95% CI 1.741–4.667, P < 0.0001), without distant metastasis (adjusted OR = 2.591, 95% CI 1.569–4.278, P = 0.0002) and with distant metastasis (adjusted OR = 17.478, 95% CI 4.916–62.144, P < 0.0001), with invasion (adjusted OR = 6.040, 95% CI 3.083–11834, P < 0.0001) and without (adjusted OR = 2.220, 95% CI 1.288–3.826, P = 0.0041), node infiltration (adjusted OR = 5.652, 95% CI 3.154–10.128, P < 0.0001), luminal A type (adjusted OR = 2.839, 95% CI 1.529–5.269, P = 0.0009), luminal B type (adjusted OR = 2.416, 95% CI 1.143–5.106, P = 0.0208), Her2 positive type (adjusted OR = 4.509, 95% CI 2.046–9.939, P = 0.0002), ER positively expressed (adjusted OR = 3.397, 95% CI 1.746–6.542, P = 0.0003) and negatively expressed (adjusted OR = 2.642, 95% CI 1.536–4.543, P = 0.0004), PR positively expressed (adjusted OR = 3.313, 95% CI 1.722–6.372, P = 0.0003) and negatively expressed (adjusted OR = 2.672, 95% CI 1.553–4.597, P = 0.0004), Her2 positively expressed (adjusted OR = 2.662, 95% CI 1.508–4.699, P = 0.0007) and negatively expressed (adjusted OR = 3.176, 95% CI 1.740–5.795, P = 0.0002), and Ki67 expressed cells ≥ 10% (adjusted OR = 3.018, 95% CI 1.494–6.097, P = 0.0021) and < 10% (adjusted OR = 2.863, 95% CI 1.687–4.860, P < 0.0001).

The rs2449512 polymorphism exhibited a more substantial risk association among patients aged older than 53 years (adjusted OR = 8.251, 95% CI 1.826–37.289, P = 0.0061), younger than 53 years (adjusted OR = 3.267, 95% CI 1.259–8.477, P = 0.0150), with tumor size smaller than 2 cm (adjusted OR = 5.770, 95% CI 2.495–13.384, P < 0.0001) and larger than 2 cm (adjusted OR = 3.239, 95% CI 1.203–8.724, P = 0.0201), possessing a family history (adjusted OR = 5.987, 95% CI 1.208–29.667, P = 0.0284) or no family history (adjusted OR = 4.375, 95% CI 2.003–9.555, P = 0.0002), being in post-menopause (adjusted OR = 8.180, 95% CI 2.008–33.327, P = 0.0034) or pre-menopause (adjusted OR = 3.380, 95% CI 1.387–8.240, P = 0.0074), presenting clinical stage 1 (adjusted OR = 5.648, 95% CI 1.139–28.004, P = 0.0340), clinical stage 2 (adjusted OR = 4.699, 95% CI 2.002–11.027, P = 0.0004) or clinical stage 3 (adjusted OR = 4.117, 95% CI 1.454–11.660, P = 0.0077), characterized by a high pathological grade (adjusted OR = 4.621, 95% CI 2.152–9.922, P < 0.0001), without distant metastasis (adjusted OR = 4.559, 95% CI 2.218–9.766, P < 0.0001), invasion (adjusted OR = 10.516, 95% CI 3.929–28.143, P < 0.0001) or without (adjusted OR = 3.338, 95% CI 1.422–7.835, P = 0.0056), node infiltration (adjusted OR = 6.128, 95% CI 2.367–15.866, P = 0.0002) or without (adjusted OR = 3.886, 95% CI 1.653–9.136, P = 0.0019), and luminal A type (adjusted OR = 4.851, 95% CI 1.910–12.322, P = 0.0009), Her2-positive type (adjusted OR = 5.005, 95% CI 1.514–16.537, P = 0.0083) and basal-like type (adjusted OR = 9.733, 95% CI 3.240–29.243, P < 0.0001), ER negatively expressed (adjusted OR = 6.871, 95% CI 2.775–17.014, P < 0.0001) and positively expressed (adjusted OR = 3.550, 95% CI 1.471–8.570, P = 0.0048), PR negatively expressed (adjusted OR = 6.686, 95% CI 2.704–16.531, P < 0.0001) and positively expressed (adjusted OR = 3.599, 95% CI 1.490–8.695, P = 0.0044), Her2 negatively expressed (adjusted OR = 5.924, 95% CI 2.595–13.524, P < 0.0001) and positively expressed (adjusted OR = 2.922, 95% CI 1.034–5.254, P = 0.0430), and Ki67 expressed cells < 10% (adjusted OR = 3.045, 95% CI 1.240–7.475, P = 0.0151) and ≥ 10% (adjusted OR = 9.013, 95% CI 3.677–22.094, P < 0.0001).

### Haplotype analysis of three *MTDH* gene SNPs correlated with IDC susceptibility

We further investigated the potential association between the haplotypes of the three *MTDH* gene SNPs and the risk of IDC. As illustrated in Table 4, the wildtype allele TGA was designated as the reference group. Compared to the reference haplotype TGA, the following haplotypes were found to be significantly associated with an increased risk of IDC: TAA (adjusted OR = 6.983, 95% CI 3.788–12.874, P < 0.001), TAG (adjusted OR = 2.392, 95% CI 1.007–5.682, P = 0.048) and TGG (adjusted OR = 1.584, 95% CI 1.150–2.181, P = 0.005).

**Table 4:** Association between inferred haplotypes of the MTDH gene and IDC risk

Haplotypes^a^	Cases (n = 300)	Controls (n = 565)	Crude OR (95% CI)	*P*	Adjusted OR^b^ (95% CI)	*P*
	No.%	No.%				
TGA	404 (23.35)	883 (51.04)	1.000		1.000	
CGA	45 (2.60)	98 (5.66)	1.004 (0.692–1.456)	0.985	0.986 (0.679–1.431)	0.940
TAA	45 (2.60)	14 (0.81)	7.025(3.812–12.946)	< 0.001	6.983 (3.788–12.874)	< 0.001
CAA	6 (0.35)	8(0.46)	1.639 (0.565–4.755)	0.363	1.585 (0.545–4.607)	0.398
CAG	1 (0.06)	0	> 999.999 (< 0.001, > 999.999)	0.979	> 999.999 (< 0.001, > 999.999)	0.978
TAG	11 (0.64)	10 (0.58)	2.404 (1.013–5.707)	0.047	2.392 (1.007–5.682)	0.048
TGG	76 (4.39)	103 (5.95)	1.613 (1.172–2.218)	0.003	1.584 (1.150–2.181)	0.005
CGG	12 (0.69)	14 (0.81)	1.873 (0.859–4.087)	0.115	1.810 (0.828–3.956)	0.137

^a^The haplotypes order was rs1311, rs16896059, and rs2449512

^b^Obtained in logistic regression models with adjustment for age

## Discussion

In the present case–control study, involving 300 IDC cases and 565 healthy controls from Eastern Chinese populations, we investigated the potential association between *MTDH* gene polymorphisms and the risk of IDC. Our findings provide evidence that three polymorphisms, namely rs1311 T > C, rs16896059 G > A, and rs2449512 A > G, were associated with an increased susceptibility to IDC. Notably, our study is the first to establish the association between *MTDH* polymorphisms and IDC susceptibility.

MTDH plays a crucial role in various stages of carcinogenesis and serves as an oncogene in multiple cancers. In breast cancer, MTDH acts as a mediator for numerous non-coding RNAs. For instance, the long non-coding RNA (lncRNA) FAM83H-AS1 facilitates the progression of triple-negative breast cancer through binding miR-136-5p to increase MTDH expression [27]. Knockdown of lncRNA TP73-AS1 inhibits in vitro breast cancer cell carcinogenesis by targeting miRNA-125a-3p to suppress MTDH levels [28]. LINC00707 directly targets MTDH to inhibit breast cancer by sponging miR-876 [29]. Furthermore, anti-cancer agents can target MTDH to suppress breast cancer. Lobaplatin inhibits cell proliferation and induces apoptosis by downregulating MTDH in breast cancer [30]. The tumor suppressor FBXW7 also hinders breast cancer proliferation and promotes apoptosis by degrading MTDH [31]. Overexpression of MTDH is associated with doxorubicin sensitivity of breast cancer [32]. Furthermore, the expression of MTDH is prognostically linked to diverse molecular subtypes among patients with breast cancer following therapeutic intervention. Li Y et al., employed Affymetrix microarrays to identify genes that are differentially expressed between estrogen-treated parental cells and those deficient in MTDH. Subsequently, they determined that MTDH and ERα interact within the nucleus under estrogenic treatment to regulate gene expression [33]. Chu PY, et al. demonstrated that MTDH serves as an independent prognosticator of inferior outcomes in patients with ER-negative or PR-negative breast cancer [14]. Elevated MTDH expression predicts a better prognosis for HER-2 positive breast cancer patients following combined therapy of neoadjuvant chemotherapy and trastuzumab [15]. Despite numerous studies exist regarding the function of *MTDH* in breast cancer, the association between *MTDH* polymorphisms and breast cancer risk remains unreported. However, one study did reveal a negative association between the *MTDH* (− 470G > A) polymorphism and ovarian cancer susceptibility [21].

In the current study, genotyping was performed on three SNPs of *MTDH*, rs1311, rs16896059, and rs2449512. The results revealed a significant association between the genotypes of rs1311 T > C, rs16896059 G > A, and rs2449512 A > G with an increased risk of IDC. Our findings suggested that the presence of the rs1311 C allele, rs16896059 G allele, and rs2449512 G alleles exacerbated the IDC risk in Eastern Chinese women. Furthermore, all three polymorphisms were found to contribute to elevate IDC risk in women aged 53 years or younger, without a family history, with pre-menopause status, clinical stage 2, without distant metastasis or invasion. These associations were observed specifically in patients with Her2-positive type, ER positive, PR positive, and Ki67 cells greater than 10%. However, the effects of rs16896059 and rs2449512 on IDC risk were more prominent in patients with tumor size larger than 2 cm, post-menopause, clinical stage 3, low pathological grade, invasion, node infiltration, ER negative, PR negative, Her2 negative, and Ki67 cells less than 10%. Additionally, these results indicated that the genotype rs1311 A > G could serve as a reference for IDC subtyping and therapeutic decision-making. Based on previous research [34], haplotypic association studies involving multiple SNPs have been shown to significantly enhance gene mapping power compared to single SNP studies when it comes to identifying disease-causing genes. Consequently, we investigated whether haplotypes composed of *MTDH* gene polymorphisms rs1311, rs16896059, and rs2449512 were associated with IDC risk. Our findings revealed that these variants could interact with each other to influence the risk of IDC.

The effect and function of these three selected SNPs in *MTDH* gene has not been reported. Since rs1311 and rs2449512 are located in the 3ʹUTR of *MTDH* gene, they were predicted to affect miRNAs binding. Non-coding RNAs regulate the expression and function of MTDH by affecting their binding to miRNAs in breast cancer. For instance, circHIPK3 in breast cancer-derived exosomes promotes angiogenesis in the tumor microenvironment through elevating the expression of MTDH by sponging miR-124-3p [35]. OTUD6B-AS1 facilitates paclitaxel resistance by sponging miR-26a-5p to upregulating MTDH, then promoting autophagy and genome instability [36]. MiR-9-3p enhances the drug resistance of gemcitabine by directly targeting MTDH [13]. A specific H3K79 methyltransferase DOT1L increase the MTDH expression by increasing H3K79me3 levels on its promoter to promoting angiogenesis in triple-negative breast cancer [37]. As rs16896059 is located in the promoter of *MTDH* gene, its polymorphism might regulate transcriptional activity. In the future study, we need to perform experiments to verify the effects of these three SNP polymorphisms on the expression and function of MTDH.

There are still several limitations in this study. Firstly, the sample size was inadequate. Secondly, being a retrospective study, it inevitably resulted in information bias and selection bias. To mitigate these biases, we employed frequency matching of cases and controls based on age and BMI. Thirdly, the samples were recruited from a single center, potentially introducing unavoidable selection bias. This study primarily focused on the analysis of genetic variations associated with IDC susceptibility. However, crucial information such as environmental factors, gene mutations, breastfeeding, and lifestyle was unavailable for analysis. Lastly, the relationship between *MTDH* gene polymorphisms and the prognosis of IDC was not examined in the current study.

In summary, our findings demonstrated a significant association between polymorphisms rs1311 T > C, rs16896059 G > A, and rs2449512 A > G in the *MTDH* gene and an increased risk of IDC in Eastern Chinese women. Further investigation was warranted to elucidate the biological function of these *MTDH* gene risk SNPs in the etiology of IDC. Our results indicated that polymorphisms in the *MTDH* gene were linked to a heightened susceptibility to IDC. These findings suggested that *MTDH* gene polymorphisms had potential as biomarkers for assessing IDC susceptibility.

## Data Availability

The datasets generated during and/or analyzed during the current study are available from the corresponding author on reasonable request.
